# The stigma of schizophrenia from patients' and relatives' view: A pilot study in an Italian rehabilitation residential care unit

**DOI:** 10.1186/1745-0179-3-23

**Published:** 2007-10-29

**Authors:** Chiara Buizza, Beate Schulze, Elena Bertocchi, Giuseppe Rossi, Alberto Ghilardi, Rosaria Pioli

**Affiliations:** 1 Clinical Psychology Section, Faculty of Medicine, University of Brescia, Brescia, Italy; 2 Public Mental Health Research Group, University of Zurich, Department of General and Social Psychiatry, Zurich, Switzerland; 3 Psychosocial Rehabilitation Unit, IRCCS "Centro S. Giovanni di Dio" Fatebenefratelli, Brescia, Italy; 4 Psychosocial Rehabilitation Unit, IRCCS "Centro S. Giovanni di Dio" Fatebenefratelli, Brescia, Italy; 5 Clinical Psychology Section, Faculty of Medicine, University of Brescia, Brescia, Italy; 6 Psychosocial Rehabilitation Unit, IRCCS "Centro S. Giovanni di Dio" Fatebenefratelli, Brescia, Italy

## Abstract

**Objective:**

To identify the constituent elements of the stigma from the perspective of those having first-hand experiences of it.

**Methods:**

Subjective experiences of stigma were explored in six focus groups: three with people suffering from schizophrenia and three with patients' relatives. Focus group sessions were tape-recorded, transcribed and analyzed by means of an inductive method, forming categories from the texts, as a basis for coding. Analysis aimed at establishing a typology of stigmatization experiences from the spoken words of the focus group participants.

**Results:**

Four dimensions of stigma were identified: access to social roles; internalization of stigma; quality of mental health services, public image of mental illness.

**Conclusion:**

The most frequently found topics concerned experiences of marginalization and discrimination that people with schizophrenia experience in their daily life. These results mirror the findings of similar studies obtained in other cultural contexts.

## Background

The living conditions of people with schizophrenia do not only depend on the severity of the illness, but also on the level of their acceptance in the community. Despite recent treatment advances, those suffering from schizophrenia face a considerable stigma that limits access to treatment and hinders their full integration into society [1-7]. Stigma was conceptualized as an attribute that is deeply discrediting and makes the person carrying it different from others and of a less desirable kind. However, individuals with schizophrenia are not the only ones to be stigmatized. The stigma is also conferred upon relatives, close friends and all those who come into close contact with the mentally ill, including mental health professionals [8]. Most previous studies sought to understand stigma by studying public attitudes and beliefs [9-11]. Based on these findings, efforts to combat stigma have primarily been focused on changing these stigmatizing attitudes towards people with schizophrenia. Little research has been done to explore first person accounts of stigma as experienced by patients, relatives or mental health practitioners. However, recent stigma research has begun to enquire the subjective perspective [12-14], using a qualitative approach. Results show that psychiatric patients are exposed to stigmatization in many ways and suggest that efforts against stigma should not only be directed at improving community attitudes, but that these programs should also address patients and their relatives [15].

### Aims of the study

The present study was conducted as part of a larger international anti-stigma effort, the World Psychiatric Association's Global Program to Fight Stigma and Discrimination Because of Schizophrenia [16-18], which aims to dispel the myths and misunderstandings surrounding schizophrenia. Specific objectives of this study are to identify and to understand the stigma from the perspective of people with schizophrenia and their relatives in our local context, in order to collect concrete suggestions for reducing stigmatization because of schizophrenia. For this aim we used the focus groups technique, a qualitative method which has been described as particularly effective for collecting data in a limited time and allowing in-depth analysis, while also valuing participants' interactions [19-23]. There has been a renewed interest for this technique and related qualitative approaches over the last decade, both in the social sciences, and, more recently, also in psychiatry [e.g. [24-26]]. Qualitative methods are particularly suited for exploring subjective views and perceptions and for understanding concrete everyday experiences of social phenomena such as stigma [27], which were explicit aims to the present research. Gaining an insight in the experiences of those at the receiving end of stigmatization is a central prerequisite for developing needs- and evidence-based inventions to effectively fight stigma [28].

## Methods

### Participants and data collection

Stigma experiences were explored in six focus groups: three with patients and three with their relatives. Total sample size was 48: 26 users and 22 relatives (8 mothers, 4 fathers, 6 sisters, 3 brothers, 1 husband). 57% of the sample was male. All patients were in treatment at a psychiatric rehabilitation unit: 75% were inpatients, 25% outpatients. Mean age was 44.5 years (± 8.88). Twelve subjects (4 patients and 8 relatives) refused to participate in the study. Participants were recruited through letters of invitation, which were distributed at the hospital through the researchers participating in the anti-stigma program and in co-operation with the *Mental Health Alliance *of Brescia (Italy). Patients were eligible for the study if they had an ICD-10 diagnosis of schizophrenia. All participants gave a written informed consent. The focus groups sessions were facilitated by a team of two psychologists trained for this purpose. They used a question guide (Table 1) translated from focus group guidelines used in previous research [12] on the stigmatization experiences of people with schizophrenia and adopted in the framework of the WPA's global effort against stigma [29]. Discussions were tape-recorded and transcribed.

**Table 1:** Question guide used in the focus group sessions

TOPIC 1: STIGMATISATION EXPERIENCES
*Opening question:*
What has changed for you after you first developed schizophrenia? Tell me *concrete incidences and stories *that you experienced!
*Further questions (alternative):*
Were there situations in which you felt excluded or misunderstood? [if nec., probe: when? where? can you describe? other situations than already described?]
Did you tell other people that you had schizophrenia? [if nec., probe: whom? when? why? why not?]
How did people around you react when they found out you had schizophrenia? [if nec., probe: withdrawal, interest, gossip, support?]
TOPIC 2: CAUSES OF STIGMA
*Questions (alternative):*
Why, do you think, do people react in this way?
Why do people think in this way about people with schizophrenia?
In your opinion, where do these stereotypes/views come from? [if nec., probe: media, history, everyday language, lack of information?]
TOPIC 3: SUGGESTIONS FOR ANTI-STIGMA INTERVENTIONS
*Questions (alternative):*
What should be done about negative stereotypes/discrimination because of schizophrenia?
How would you like people to react to the fact that you have schizophrenia?
How, do you think, could these situations (described earlier) be avoided/improved?
What kind of information would be important?
Who/which groups in particular should be addressed?

### Statistical Analysis

Recordings from the focus group sessions were transcribed verbatim and analyzed by means of qualitative content analysis as proposed by Mayring [30,31]. Two trained investigators carried out coding independently, before deciding on the final coding scheme. Inter-rater reliability was checked on a sample of 100 statements and proved to be satisfactory (Cohen's Kappa = 0.80).

A separated analysis for patient and relative groups was conducted. First, a descriptive summary of the main information of each focus group was prepared based on field notes from the session. Secondly, categories were formed inductively from the transcripts for establishing a typology of stigmatization experiences [19,32,33], reflecting a grounded theory approach [34]. In the process, relevant passages of text with regard to the aims of the study were clustered under the general categories [32]. The final goal of analysis was to achieve saturation [33]. To avoid an exaggerated focus on single fragments of text [35], interactions in the course of the group session were taken into consideration [36]. In the course of the analysis, new codes were added to the coding system, or linked to existing codes whenever relevant. In order to estimate the weight of the single categories, the frequency of mentions in each final category was calculated. Analysis was supported by using WinMax, a software package for qualitative data analysis [37].

## Results

**Figure 1:**
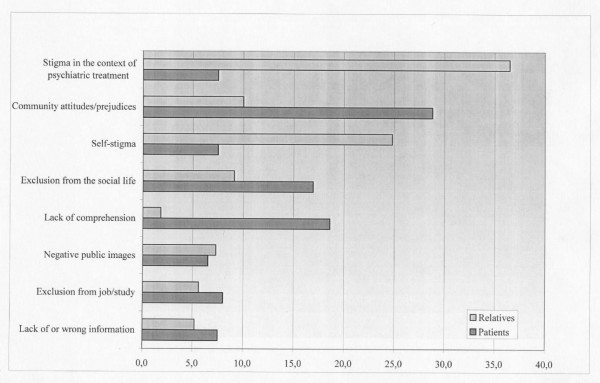
Experiences of stigmatization.

The sample reported 428 concrete cases of stigmatization (Figure 1): 198 experiences were described by patients and 230 by relatives. The largest number of statements concern *stigma experienced in the context of psychiatric treatment*. Relatives strikingly often reported stigmatizations occurring in their relationship with mental health professionals: 36.5% of all mentioned incidences, against only 7.5% of the patients. Relatives reported lack of collaboration with families during the treatment, inadequacy of service structures and shortage of effective treatments. Both patients and their relatives further reported concrete cases of discrimination and unwarranted coercive measures in the context of psychiatric treatment.

The second most frequently reported stigmatization experiences are related to the *negative attitudes and prejudices by the community*. Rejection by the public was more frequently reported by patients (28.8%) than by their relatives (1.3%).

The third most important feature of stigma is *self-stigmatization*. The effects of stigma on patients' self-perception are described by one fourth of the relatives (24.8%), but by only 7.5% of people with schizophrenia. Further, those interviewed described situations of *social exclusion*, loss of friends, relatives and colleagues (16.9% of the patients and 9.1 % of their relatives). Moreover, 18.6% of users experiences, compared to 1.3% of relatives accounts, concern the *lack of comprehension *for their situation they meet in everyday life. This seems to be fostered by the *negative representation of mental health problems offered by the media*, which was described as stigmatizing by 6.5 % of the patients and by 7.3% of their relatives. In 8% of the users' reports and in 5.6% of the relative's accounts, it was moreover stated that the fact that one suffers from a mental illness hinders the *access to an occupation *and makes it difficult to maintain a job. Finally, both patients (7.5%) and their relatives (5.2%) claim that there is a *lack of knowledge *about mental disorder that often leads to a biased view of the illness and of its treatment, especially regarding the responsibility for the illness which is often attributed to the patients themselves and/or to their relatives.

### Dimensions of stigma experiences

**Figure 2:**
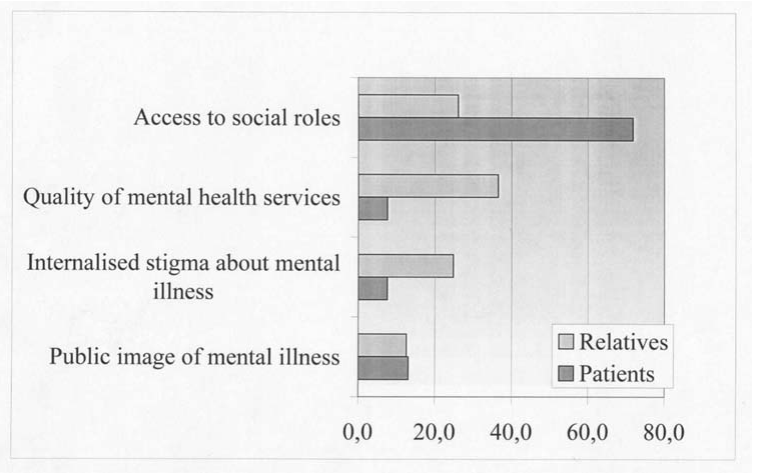
Dimensions of stigma.

From the patient and relative groups' statements we identified four dimensions of stigma (Figure 2). These overall themes apply equally to patients and relatives, while concrete experiences within the stigma domains both overlap and vary between the two groups. Differences and similarities will be described below and illustrated by verbatim quotes from focus group participants (Table 2).

**Table 2:** Stigma Experiences of Patients and Relatives: Verbatim Quotes

ACCESS TO SOCIAL ROLES
*Quote 1 (R., patient, female)*: "... what really made me suffer more was the abandonment and the indifference on the part of my friends ... slowly I have lost them. I had only one friend left; we grew up together; we had always been like brothers. I would never have thought (I'd ever lose him), but when I told him (about the illness), he disappeared."
*Quote 2 (C., relative, sister)*: "... I can tell my experience. When I was 37 years old and my brother came back home, I realized that his presence prevented me to have close relationships with my friends. Then I went to live away from my family."
QUALITY OF MENTAL HEALTH SERVICES
*Quote 1 (P., patient, female)*: "They are going to open a sheltered apartment in street G. I ask myself: Does it have to be in the industrial area at the outskirts of this town? There aren't any services, facilities or stores, there!"
*Quote 2 (S., relative, mother)*: "... doctors are superficial in administering the treatments; they change them frequently. They are superficial in giving diagnoses, often they do not know to respond to the problems related with the disease, or they do not know how to explain the illness. Psychiatrists in the public service change continuously and you must tell each new one the whole story and all the problems all over again. Every doctor, then, makes his own diagnosis and changes the treatment... However, patients are not guinea pigs for experiments!"
INTERNALISED STIGMA ABOUT MENTAL ILLNESS
*Quote 1 *(A., *patient, male)*: "It's true that stigma comes to us from outside, but, in my opinion, a lot of stigma comes to us also from our way of relating to the illness. Most people believe that mental illness is something different from other illnesses such ad heart disease, liver disease and eye problems. But mental illness is a disease that can be cured with the right medication and by accepting the psychiatrist as a specialist that cures that disease .... by accepting this we take a step towards improvement."
*Quote 2 (D.., relative, father)*: "We are the first people who are convinced of this, that those with mental illness are aggressive. This is the problem. This leads to a point where it triggers a mechanism through which we no longer deal with persons, but only with sick ones."
PUBLIC IMAGE OF MENTAL ILLNESS
*Quote 1 (T., patient, male)*: "In my opinion, the prevailing factor is the (public's) fear of something which is little known, such as mental illness ... the information about it does not circulate."
*Quote 2 (M., relative, mother)*: "When there is someone who kills, or hurts another person, the television and the newspapers always claim that it was a schizophrenic's fault, that he or she has had a madness attack ... Who is making all these diagnoses? I wonder if the journalists know what they are talking about."

#### Access to social roles

This dimension refers to the discriminating attitudes that patients meet in their everyday life. Patients and their relatives identified the cause of these attitudes in a lack of understanding and in the community prejudices. Users report to feel neglected, to provoke fear and to be permanently recognized as "different". Many experiences of retreat, or loss of friends, are reported. Patients further feel that they are ridiculed, rejected and watched with curiosity. In patients' and relatives' view, the most common public prejudice has to do with the idea that the illness is under control of the sick persons themselves. Almost all patients agree in thinking that these attitudes are determined by the fact that their friends and family cannot understand mental illness. Patients further state that people have difficulty recognizing schizophrenia as an illness like any other. All the above is perceived as leading to people clustered into categories providing justifications for the community in its closing off any relationship with those suffering from schizophrenia. The assumption of negative community attitudes then results in the social withdrawal on the part of patients and their relatives themselves.

Exclusion also frequently takes place through discrimination experienced in the workplace. Patients are often disheartened because they believe that professional opportunities are limited once their illness becomes public. Patients and their relatives see stigma reflected in the fact that actual job prospects for those with schizophrenia are limited to little rewarding positions in sheltered settings. Patients not only have troubles while job hunting, but also in keeping a job once employed. Patients and relatives alike report that employers become suspicious whenever a person is recognized as being affected by any mental illness and that their colleagues often reject and discriminate them. Patients also report having trouble when looking for a friend or a partner, or when trying to maintain an existing relationship. Relatives' social ties are affected by the stigma of schizophrenia, too: they experience abandonment by their relatives and friends who feel uneasy with them.

#### Quality of mental health services

This dimension refers to the perceived inadequacy of treatments and institutions for the mentally ill; to the lack of, or difficult collaboration between mental health practitioners, patients and family members; and to the lack of legislation protecting the rights of mentally ill people and their relatives. Experiences in this domain were described by those relatives whose contacts with mental health services had often been a frustrating experience. In general, relatives appear to report negative impressions concerning the quality of services and treatments more easily than patients do. Patients and their relatives agree in complaining about the low number of services and structures. Caregivers also believe that the public mental health service is too little specialized and not well equipped to provide adequate care. Services are perceived merely as "...*quick fixes*", lacking any long-term effectiveness, with involuntary commitment being perceived as the worst, because of its highly stigmatizing effects. At the same time, relatives believe that involuntary commitment is often an unavoidable last resort, as usually nobody intervenes before the illness aggravates up to a dangerous point. Patients and their relatives, moreover, state that mental health services are understaffed, and therefore are unable to provide effective treatment and care. Not only therapies are considered inadequate, but those questioned also reported situations of inadequate behavior by the psychiatrists. Moreover, relatives state that they are little involved in the treatment of their family member. They believe that doctors do not listen to them and that they underestimate their point of view and their experience regarding early signs of crisis. In addition, no support is given for handling the caregiver burden. Patients, too, report that they do not to feel understood by doctors: they feel lonely during their time at the hospital and believe that they are not listened to, or considered seriously.

#### Structural discrimination

In addition to negative experiences regarding the availability and quality of mental health services, patients and their relatives reported experiences of legal discrimination. Mental illness is hardly considered by the political and legislative world, which does not take into account every day problems faced by those with schizophrenia and their families and treats patients without considering their personal history. Relatives are critical of law 180 (law that reformed psychiatric assistance in Italy and enacted a new system of community-based services, progressively abandoning psychiatric hospitals) that, in their opinion, has closed down part also helpful mental health services. As a result, relatives feel alone in their management of patients.

#### Internalized stigma of mental illness

This dimension refers to the alienation of patients and their relatives because of the process of internalization of stigma. Stigma, prejudices and discrimination are also present among patients and their relatives. Many respondents have learnt negative opinions on mental illness and eventually attribute them to themselves and/or their ill relative. This may lead to self-induced discrimination: people with schizophrenia feel discouraged from seeking employment and reinforce their own social isolation to avoid stigmatizing reactions. This circuit is not only aggravated by the fact that it affects patients' self image and identity, but it also diminishes the awareness of their civil rights, both on the part of patients themselves and their relatives.

#### Public image of mental illness

This dimension mainly refers to the lack of and or wrong information spread by the media on mental illness. Many patients perceive the misleading, or lacking information on their illness among the public to be at the root of their social isolation and of the distrust that they face from other people. The public bias in information presents a negative picture, contributing to perpetuate false beliefs among the population, fostering fears and social rejection. People with schizophrenia are still depicted by mass media as dangerous and strange, often in connection with brutal crimes. Moreover, in patients and relatives views, the superficiality and carelessness with which psychiatric terms are used in everyday language feed a biased idea of schizophrenia in the community. Finally, patients and relatives deplore a lack of information campaigns to increase awareness and promote mental health.

### Stigma and self-stigmatization – Mechanisms in the relationship between people with schizophrenia and their relatives

Part of the relatives make patients feel guilty by holding them responsible for behaviors related to their illness. Family members are ashamed of their relatives and they try to hide the fact that someone in their family suffers from a mental illness, fearing negative reactions from the community. The majority of relatives believe that people suffering from a mental illness are violent, dangerous and incurable. They therefore react with fear and place little confidence in them, considering them malicious and lacking willpower. Relatives also believe that having a relative with a mental disorder is a condition that destroys the family, a process triggered by illness-related difficulties, changes in the relationship with the ill relative and substantial caregiver burden resulting from it. Consequently, relatives call for more service structures. For some, this extends as far as wishing "*to lock them up*", as they believe that people with mental illness cannot live with "normal" persons. It is often evident that, in the relationship with their ill relative, carers feel like having to do with "*something strange and incomprehensible*". At the same time, relatives recognize that stigma partially starts from them; from their attitude that wrongly holds patients responsible for their condition. At the same time, patients equally show understanding for stigmatizing attitudes among their relatives. Finally, patients recognize that some of their behaviors are not easy to understand and they also think that the feelings of shame and the illness-concealing attempts on the part of their relatives are – to some extent – justifiable.

## Discussion

Before discussing the results in detail, it is necessary to highlight that the sample cannot be considered representative of people with schizophrenia and their relatives, since we did not apply a randomized inclusion protocol. For this reason, it does not allow to draw generalizable conclusions.

The data collected highlight that, in the Italian context too, the views of people with schizophrenia and their relatives on stigma broadly overlap with the findings of similar studies in the general population. Our results also mirror the findings of studies on subjective stigma experiences obtained in other cultural contexts [38-41], using similar methodologies [12,14,42]. The similarities of findings confirm the fact that stigma and discrimination are a universal phenomenon, with the concrete experiences made by patients and relatives being much alike across cultures. However, our results highlight that there are differences as well. In the Italian context, people with schizophrenia feel that barriers in accessing social roles are the most discriminating aspect of their stigma-related experiences. This might be because 75% of our sample is represented by inpatients of a rehabilitation residential care unit. Their long illness and hospitalization history might partially explain this focus on stigma as an obstacle to participation in society. Similarly, differences such as the prevalence of self-stigma in our study, which was not found elsewhere, and respondents' inability to envisage stigma coping strategies and avenues for positive change, may be attributed to the different composition of samples and to variations in opportunity structures between life in the community and in a segregated setting.

Stigmatization in the workplace and the related denial of access to job are the most important experiences of social exclusion. These experiences are recognized as the main factor producing and maintaining a high rate of unemployment among those with schizophrenia. This is particularly striking when one considers that recent studies have produced evidence that 30–40% of persons with serious mental illness are able to work [43-48]. Setting up programs directed towards modifying the attitudes of employers, as well as greater attention to the employment-related training of social workers could constitute useful instruments to improve labor market access for people with schizophrenia. These programs should help convincing employers that people with schizophrenia are highly motivated and can provide important contributions. At the same time, people with schizophrenia might need specific support in re-entering the labor market, such as *Individual Placement and Support (ISP) Programs *[49,50]. Better integration in the labor market has been shown to improve clinical outcomes [44,45,47] and reduce the risk of re-hospitalization [51].

Another previous finding confirmed by our analyses is that stigma and negative public attitudes are shared by patients and their families. Therefore, while they continue to consider stigma as a central obstacle to their integration into the community, they themselves contribute to this process by accepting public stereotypes as applicable to themselves. As a result, the majority of them does not confront negative reactions, lose self-esteem, isolate themselves and get worse. Our data also highlighted that both patients and relatives focus on their stigmatization experiences and on their ideas of the causes of the stigma; while hardly offering any suggestions for anti-stigma interventions. Suggestions of participants mainly concerned the improvement of information on mental health issues among the public. Participants did not seem to envisage many opportunities for positive change and found it difficult to formulate specific proposals on what could be done to improve their situation. These two findings supports the contention of labeling theory, which states that patients who accept the diagnosis of schizophrenia perceive an internal pressure to conform to the stereotypes of the illness [3,51-53].

Our results thus highlight the importance of stigma coping support and empowerment measures [54] for both patients and relatives in order to facilitate recovery. Effective anti-stigma interventions, then, should address two targets: improving attitudes and the conditions for social integration in the community and empowering people with schizophrenia and their relatives to challenge self-stigmatization and discriminatory behavior towards them [55].

Another central topic in our study was structural discrimination, having mainly to do with the quality of mental health services and legal regulations regarding mental health. The inadequacy of treatments and service structures are also a consequence of the lacking application of the Italian psychiatric reform. Even though the psychiatric reform has come into effect twenty-five years ago, it is evident that it has yet to be adequately translated into practice in many areas of Italy. There are many reasons why this law is difficult to implement, the most important being the partial dismantling of mental hospitals; the insufficient and low standard of hospitals and residential communities; as well as the severe lack of networking between services [56-59]. In addition to measures at the political and resource levels, the rehabilitation process for patients with schizophrenia requires individually tailored programs, taking account the personal levels of functioning and individual resources for recovery [60]. Moreover, education curricula for health workers do not always take into account the importance of a comprehensive treatment plans for interventions across different sectors (e.g. employment, housing, relapse prevention, etc.). In conclusion, based on the results of the present study, psychiatric services should devote more attention to mental health-related stigma, before demanding the community to do so.

## Competing interests

The author(s) declare that they have no competing interests.

## Authors' contributions

CB = she made substantial contributions to conception and design, acquisition of data, analysis and interpretation of data.

BS = she participated in the conception and design and contributed to the qualitative analysis.

EB = she performed the statistical analysis.

GR = he made substantial contribution to conception and design.

AG = he participated in the interpretation of data.

RP = she made substantial contribution to conception and design.

All authors read and approved the final manuscript.
